# TLR4-MyD88/Mal-NF-kB Axis Is Involved in Infection of HSV-2 in Human Cervical Epithelial Cells

**DOI:** 10.1371/journal.pone.0080327

**Published:** 2013-11-20

**Authors:** Hongya Liu, Kai Chen, Wenqiang Feng, Xinxing Wu, Hui Li

**Affiliations:** 1 State Key Laboratory of Virology, Institute of Medical Virology, Wuhan University School of Medicine, Wuhan, Hubei, China; 2 Shengzhen R&D Center of State Key Laboratory of Virology, Wuhan University Shenzhen Institute, Shenzhen, Guandong, China; UC Irvine Medical Center, United States of America

## Abstract

We have established an *in vitro* HSV-2 acute infection model with Human cervical epithelial (HCE cells, the primary target and natural host cells for HSV-2) to investigate the role of TLRs-mediated innate immune response to HSV-2. In current study, we found that HSV-2 infection induced activity of NF-kB reporter and expression of cytokines are TLR4-dependent using approaches with shRNA and TLR4 antagonist. Knockdown experiments demonstrated that the adaptor molecules MyD88 and Mal of the TLRs signaling pathway are required in the HSV-2 induced TLR4-dependent NF-kB activation in HCE cells. Western blot assay suggested that knockdown of TLR4 decreased the phosphorylation of IRAK1 and inhibitor of NF-kB (IkB-α) upon HSV-2 infection. Finally, decreased expression of either TLR4 or MyD88/Mal alone or both significantly abolished productions of IL-6 and IFN-β by ELISA analysis. Taken together, our results from the *in vitro* infection model reveal for the first time that there exists the pathway via TLR4-Mal/MyD88-IRAK1-NF-kB axis in human cervical epithelial cells in response to HSV-2 infection.

## Introduction

Herpes simplex virus type 2 (HSV-2) is one of the most common sexually transmitted pathogens worldwide. The estimated sero-prevalence of HSV-2 infection is more than 21% in adult population in the US. The prevalence ranges 30–80% among women in developing countries [1]. HSV-2 infection causes genital ulcer disease and is a significant co-factor in the transmission and acquisition of human immunodeficiency virus type 1 (HIV-1) [2]. HSV-2 infects the genital epithelium and can be transmitted to the central nervous system via peripheral nerve axons to the sacral ganglia to establish life-long latent infection and periodic re-activation [3]. Meningitis may occur in immuno-compromized individuals. In early pregnancy, HSV-2 can cross the placental barrier and may eventually cause fatal disseminated disease in new born [4]. Current treatments with anti-viral therapy are commonly used to control re-activation of HSV-2. However, these medications do not eliminate latent virus [5]. There is no preventative or curative vaccine available for genital HSV-2 infection [6].

The genital mucosa is the first line of defense against sexually transmitted pathogens and plays a crucial role in innate immunity [7]. The vaginal/cervical epithelial cells express a subset of Toll-like receptors (TLRs) [8], [9], which are the key pattern recognition receptors (PRRs) responsible for microorganism [10], [11]. Recent studies have shown that multiple TLRs are involved in recognition of different HSV strains and the immune response to HSV infection [12]–[19]. However, immuno-competent cells or mice model have been used in most of these studies. The attempts to define the role of NK cells, conventional dendritic cells (cDCs) and plasmacytoid DCs (pDCs) in HSV-2 infection have turned out discrepancy results [7]. This is in accord with the fact that HSV-2 does not directly infect DCs [9]. The inherent differences between rodent and human always hinder the application of murine knock-out model to human disease. It is important that the experimental immunology studies are carried out directly relevant to the diseases caused by HSV in humans [20].

HSV-2 primarily infects genital epithelium and replicates within the vaginal keratinocytes [21]. Attentions have been focused on the human genital epithelium in response to HSV-2 infection [7]. A recent paper discussed the susceptibility of primary human female genital epithelial cells to HSV-2 using an ex vivo culture model [22]. The study from the same group has exploited the anti-viral activity of human female genital epithelium in response to HSV-2, by using TLR ligands [16]. They also assessed the role of HSV-2 virion host shutoff protein on innate dsRNA antiviral pathways in human vaginal epithelial cells [23]. But little is known about the innate immune pathways of human genital epithelial cells in response to HSV-2 infection.

In previous study, we established an *in vitro* HSV-2 acute infection model with Human Cervical Epithelial (HCE) cells to investigate the role of TLRs-mediated innate immune response to HSV-2 [24]. We have shown that HSV-2 infection up-regulates TLR4 expression and activates NF-kB, and over-expression of TLR4/MD2 augments viral-induced NF-kB activation. In the current study, we found that HSV-2 infection activates innate immune response in TLR4-dependent manner in human cervical epithelial cells. The two adaptor molecules Mal and MyD88 of TLRs signaling pathways are also required for this TLR4-mediated pathway. Our results reveal for the first time that TLR4-Mal/MyD88-IRAK1-NF-kB axis is involved in response to HSV-2 infection in its primary infected genital epithelial cells.

## Results

### HSV-2 infection induced NF-kB activation and expression of cytokines in HCE cells are TLR4-dependent

**Figure 1:**
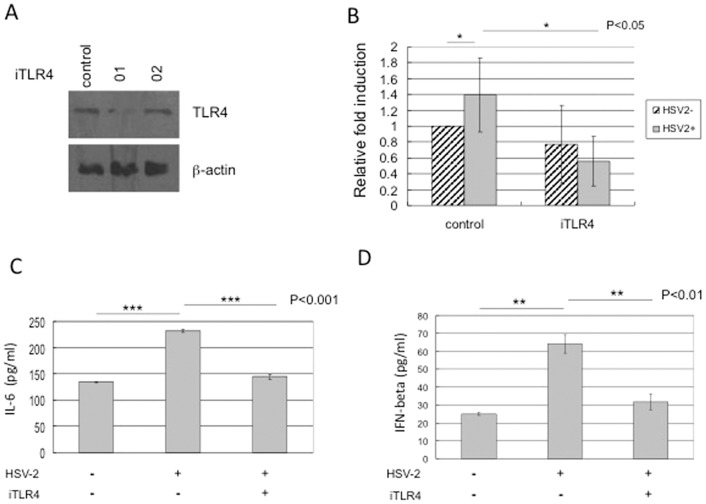
HSV-2 induced NF-kB activation and expression of cytokines in HCE cells are TLR4-dependent. (A) Knockdown of TLR4 expression. The HCE cells were transfected with two shRNA-TLR4 plasmids (No. 01 and 02) respectively or scrambled shRNA control. Forty-eight hours after transfection, the cell lysates were harvested and TLR4 level was analyzed by Western blotting, actin served as an internal loading control. The experiment was repeated three times, the representative images from one experiment are shown, No. 01 plasmid (refer to iTLR4) was sufficient to knockdown TLR4. (B) TLR4-dependent NF-kB activation by HSV-2 infection. HCE cells were transfected with vector or shRNA-TLR4 plasmids and pNF-kB-Luc and pRenilla-Luc for 48 h, then were infected with HSV-2 at 3 MOI or mock infected. The cell lysates were harvested at 4 h p.i. and analyzed for firefly luciferase activity and Renilla Luciferase activity. The normalized value from the cells with transfected control vector and without viral infection was set to 1. Knockdown of TLR4 decreased NF-kB reporter activity induced by HSV2 infection. The results are shown as means ± SD of triplicate wells. The experiment was repeated three times. * indicates p<0.05. (C) TLR4-dependent IL-6 expression by HSV-2 infection. HCE cells were transfected with shRNA-TLR4 or vector control. At 48 h after transfection, HCE cells were infected with HSV-2 at 3 MOI or mock-infected. Cell-free supernatants were collected at 4 h post-infection for measurement of IL-6 secretion by ELISA analysis. Each assay was performed in triplicate. Results are reported as means ± SD. *** indicates p<0.001. (D) TLR4-dependent IFN-β expression by HSV-2 infection. Cell-free supernatants from above transfected and infected cell cultures were collected at 1 h post-infection for measurement of IFN- β secretion by ELISA analysis. Each assay was performed in triplicate. Results are reported as means ± SD. ** indicates p<0.01.

We established an *in vitro* acute HSV-2 infection model with human cervical epithelial (HCE) cells for the study of innate immune response and found that HSV-2 infection activates transcription factor nuclear factor (NF-kB) and co-transfection with TLR4/MD2 augments this induction in HCE cells [24]. To further confirm whether TLR4 plays a role in mediating NF-kB activation by HSV-2, we screened two shRNA constructs (Figure 1A) and confirmed the knock-down efficiency with one of them (No. 01) and designated as iTLR4. HCE cells were transfected with vector or iTLR4 construct, and NF-kB reporter for 48 h, and measured luciferase activity after 4 h of HSV-2 infection or mock-infection. The NF-kB reporter activity was induced by HSV-2 (approximately 1.4 –fold) in HCE cells compared to that in mock-infected cells (p<0.05) (Figure 1B) as we previously observed [24]. Knock-down of endogenous TLR4 expression dramatically decreased the virus induced NF-kB activity (approximately 2.5-fold) compared to control (p<0.05) (Figure 1B), although the basal level of reporter activity was slightly decreased by iTLR4. The data demonstrated that HSV-2 infection activated TLR4-dependent NF-kB activation in its natural host HCE cells.

To further evaluate whether TLR4 is involved in expression of cytokines induced by viral infection, we collected the supernatants from above transfected and viral infected cell cultures at 1 h p.i. or 4 h p.i. and measured expression of IL-6 (Figure 1C) and IFN- β (Figure 1D) by ELISA assays. The basal level of IL-6 was 134.12 ± 0.23 pg/ml from mock infected cultures, while IL-6 was increased to 231.95 ± 2.79 pg/ml by 73% after viral infection (p<0.001) (Figure 1C). The level of IL-6 dropped down to same level (144.79 ± 4.67 pg/ml) of mock infected cultures after knockdown expression of TLR4 (p<0.001) (Figure 1C). We also observed the similar changes with levels of IFN- β. The basal IFN- β was 25.01 ± 0.86 pg/ml from mock infected cultures, while it was increased to 64.12 ± 5.17 pg/ml by 156% after viral infection (p<0.01) (Figure 1D). The level of IFN- β was decreased to the range (31.81 ± 4.31 pg/ml) of mock infected cultures after knockdown expression of TLR4 (p<0.01) (Figure 1D). These data suggested that TLR4 is not only play a role in activation of NF-kB reporter, but also critical to regulate expression of cytokines induced by HSV-2 infection.

**Figure 2:**
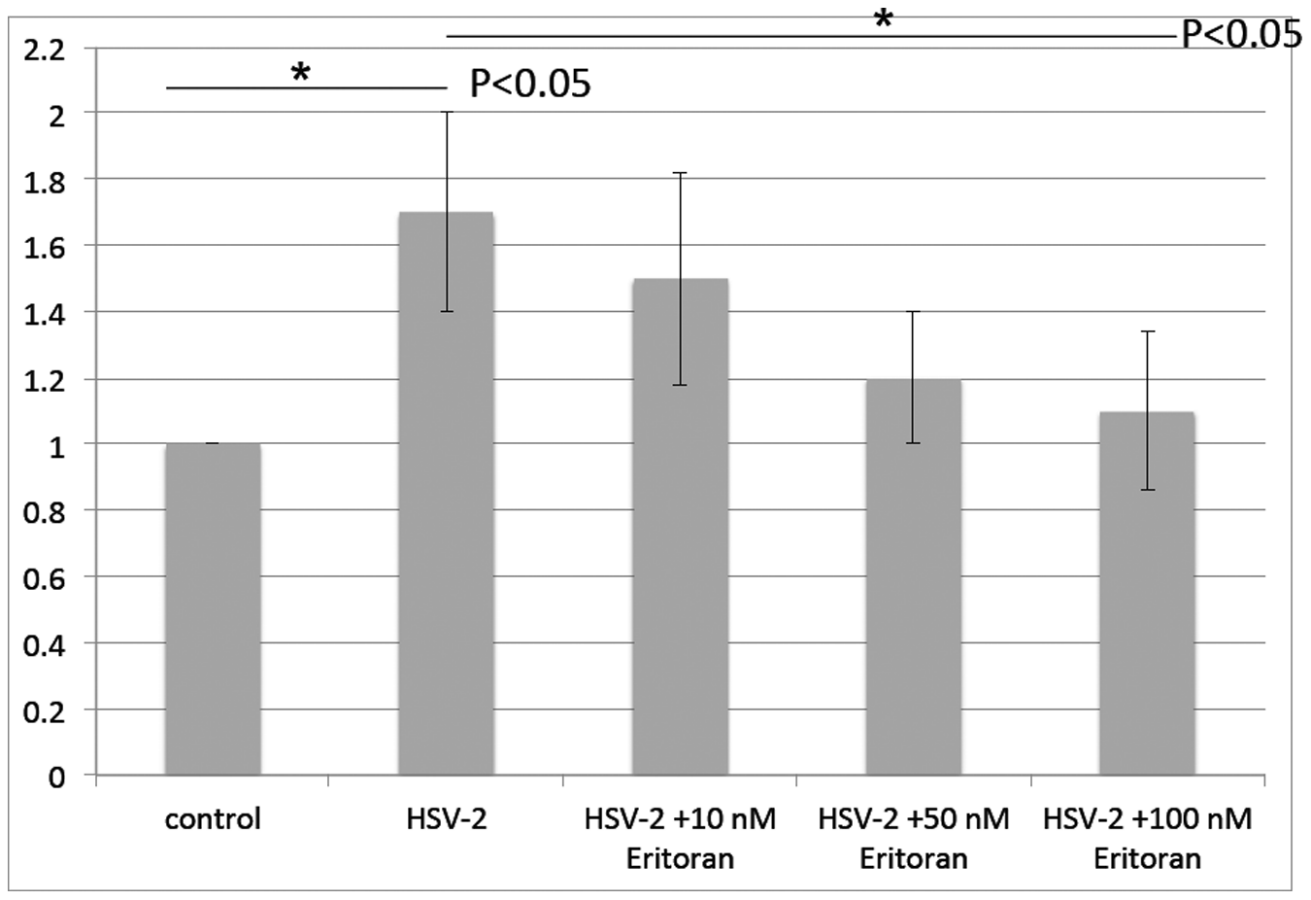
TLR4 antagonist, Eritoran, significantly decreases induction of NF-kB by HSV-2 infection. HCE cells were transfected with pNF-kB-Luc and pRenilla-Luc for 48 h, then were incubated with Eritoran at the indicated concentrations for 30 min, and then infected with HSV-2 at 3 MOI in presence of Eritoran. The cell lysates were harvested at 4 h p.i. and analyzed for firefly luciferase activity and Renilla Luciferase activity. The normalized value from the cells with transfected control vector and without viral infection was set to 1. Eritoran treatment significantly decreased NF-kB reporter activity induced by HSV2 infection in a dose dependent manner. The results are shown as means ± SD of triplicate wells. The experiment was repeated three times. * indicates p<0.05.

To strengthen the conclusive results above, we also chose the approach with existing TLR4 antagonist, Eritoran (Eritoran tetrasodium or E5564 Eisai Ins. MA). Eritoran was dissolved in endotoxin-free water and applied the NF-kB transfected HCEs before addition of HSV-2 virus. The data demonstrated that TLR4 antagonist treatment dramatically decreased induction of NF-kB activity by HSV-2 to the basal level as shown in Figure 2. The reduction of the NF-kB activity was also depending upon the concentration of Eritoran.

### HSV-2 induced TLR4-dependent NF-kB activation requires adaptor molecules: MyD88 and Mal

**Figure 3:**
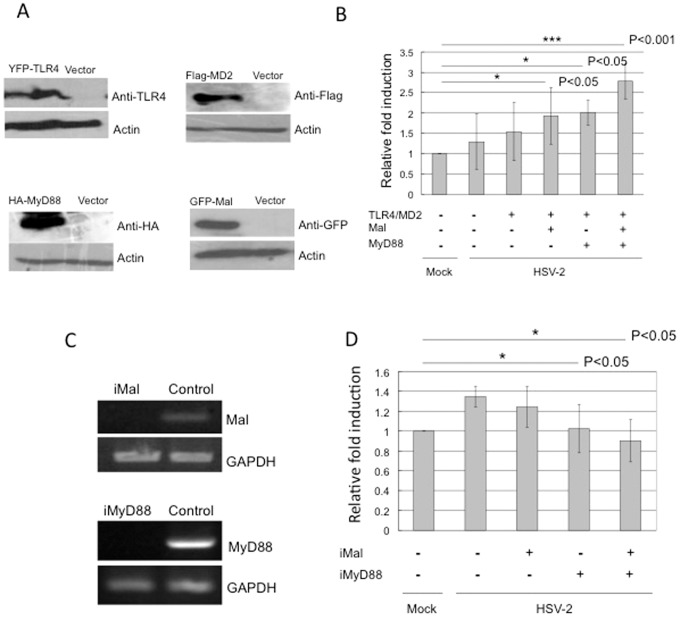
HSV-2 induced TLR4-dependent NF-kB activation requires MyD88 and Mal. (A) Confirmation of expression of TLR4, MD2, MyD88 and Mal. HCE cells were transfected with vector or TLR4, hMD2 or Mal, MyD88 expression plasmids. Twenty-four hours after transfection, expression of TLR4, hMD2, Mal and MyD88 was analyzed by Western blotting. (B) Overexpression of MyD88/Mal augments the activation of NF-kB by HSV2. HCE cells seeded in 24-well plates were cotransfected with NF-kB reporter construct together with vector or TLR4/hMD2 or TLR4/hMD2/Mal or TLR4/hMD2/MyD88 or TLR4/hMD2/Mal/MyD88 expression plasmids. A Renilla luciferase plasmid was used as a control. The transfected cells were then infected with HSV-2 at 3 MOI or mock-infected the following day. The luciferase activity was measured at 4 h p.i. and normalized to Renilla luciferase activity. The results are shown as means ± SD of triplicate wells. The experiment was repeated three times. * indicates p<0.05, and *** indicates p<0.001. (C) Confirmation of knockdown of Mal and MyD88. HCE cells were transfected with vector or siRNA-Mal (refer to iMal) or siRNA-MyD88 (refer to iMyD88). Forty-eight hours after transfection, total RNAs were collected and the expression of Mal and MyD88 was analyzed by RT-PCR. The expression of GAPDH was used as the internal control. The experiment was repeated three times, the representative images from one experiment are shown. (D) Knockdown of MyD88/Mal decreases the activation of NF-kB by HSV2. HCE cells transfected with vector or iMal or iMyD88 or iMal/iMyD88 and pNF-kB-luc and pRenilla-luc for 48 h. Cells were infected with HSV-2 (3 MOI) or mock-infection. The cell lysates were harvested at 4 h p.i. and analyzed for firefly and Renilla Luc activities. The summary of three experiments (mean ± SD) is depicted. * indicates p<0.05.

Since most TLRs need to recruit the adaptor molecule(s) (e.g. MyD88) to their receptor complex and initiate downstream signaling pathways [10]. For example, MyD88 adaptor-like (Mal) acts to bridge MyD88 to TLR4 or TLR2 complex and is capable of mediating NF-kB activation in response to bacterial infection[10], [25]. We asked whether adaptor molecules MyD88 and Mal are required in HSV-2 induced TLR4-dependent NF-kB activation in HCE cells. We first tested the effect of these two proteins on TLR4-mediated NF-kB reporter activity in response to HSV-2 infection. After confirming overexpression of MyD88 and Mal in HCE cells (Figure 3A), we co-transfected the NF-kB reporter plasmid together with TLR4/MD2, Mal/MyD88 and measured luciferase activity at 4 h after HSV-2 infection or mock-infection. We observed that MyD88 or Mal were able to augment TLR4-mediated NF-kB activity in response to HSV-2 (p<0.05) (Figure 3B), while co-expression of MyD88 and Mal enhanced TLR4-mediated NF-kB reporter activity greatly compare to that in cells expressed MyD88 or Mal alone (p<0.001) (Figure 3B). These data indicated that the adaptor molecules played a role in NF-kB activation by HSV-2 infection. More importantly, we were able to knock-down endogenous expression of MyD88 and Mal in HCE cells by siRNA duplexes iMyD88 and iMal (Figure 3C) and measure NF-kB luciferase activity in these cells in response to HSV-2. Compared with scrambled siRNA control, knock-down MyD88 or Mal reduced NF-kB activity in response to HSV-2 infection (p<0.05) (Figure 3D). The activation of NF-kB by HSV-2 was decreased further in the cells that both MyD88 and Mal were knockdown (p<0.05) (Figure 3D). Thus, our data demonstrated that adaptor molecules MyD88 and Mal were required for the HSV-2 induced NF-kB activation.

### TLR4 mediates the phosphorylation of IRAK1 in response to HSV-2

**Figure 4:**
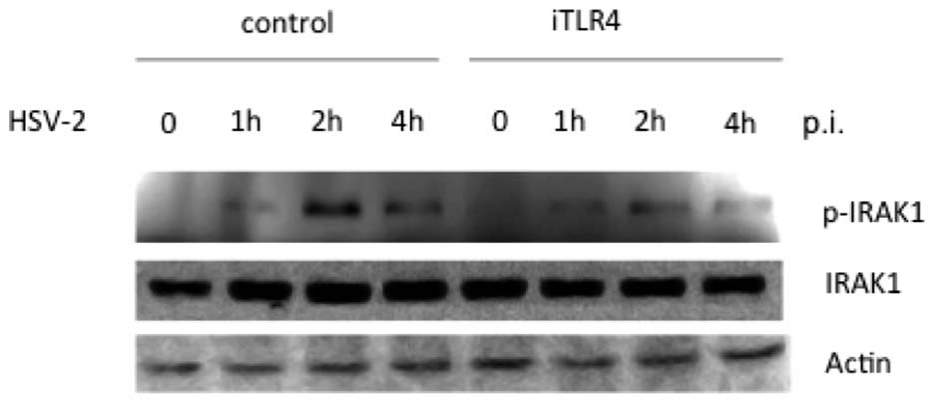
TLR4 mediates the phosphorylation of IRAK1 in response to HSV-2. HCE cells were transfected with vector or iTLR4. Forty-eight hours after transfection, cells were infected with HSV-2 or mock-infected for 0 h, 1 h, 2 h and 4 h respectively. Cell lysates were analyzed by Western blotting with antibodies against p-IRAK1, total IRAK1 and actin. Knockdown of TLR4 decreased the phosphorylation of IRAK1 by HSV2 infection. The experiment was repeated three times, the representative images from one experiment are shown.

We have shown that HSV-2 infection activates TLR4-dependent and MyD88/Mal mediated NF-kB activation in HCE cells. We wanted to assess if the key downstream components are phosphorylated in the MyD88-dependent pathway upon HSV-2 stimulation. In microphages stimulated by LPS, after the recruitment of Mal/MyD88 to the TLRs, IRAK4 is activated initially and IRAK1 is activated sequentially. IRAK1 can be a marker molecule since it undergoes hyper-autophosphorylation after phosphorylated by IRAK4 and then dissociates from the MyD88 complex and interacts with the E3 ligase TRAF6, which is capable to activate TAK1 and IKK complex [10] [26]. We hypothesized that HSV-2 infection induced the increased phosphorylated IRAK1 is dependent of TLR4. We harvested SDS lysates from HCE cells at the indicated time points after viral infection for Western blot analysis. As shown in Figure 4, IRAK1 was phosphorylated as early as 1 h after HSV-2 infection. The level of phosphorylated IRAK1 reached a peak at 2 h p.i. and declined at 4 h p.i., while in the HCE cells where TLR4 was knockdown with iTLR4 as described above, IRAK1 activation in response to HSV-2 was impaired (Figure 4). We were not able to observe an obvious peak of phospho-IRAK1 at 2 h post-infection (Figure 4). However, during the time-course of viral infection, the total IRAK1 remained unchanged (Figure 4), actin was used as the internal loading control. These data suggested that HSV-2 indeed induces the TLR4 dependent increased level of the phosphorylated IRAK1 in response to HSV-2 in HCE cells.

### TLR4 mediates the release of inhibitory IkB and activation of NF-kB signaling in response to HSV-2

**Figure 5:**
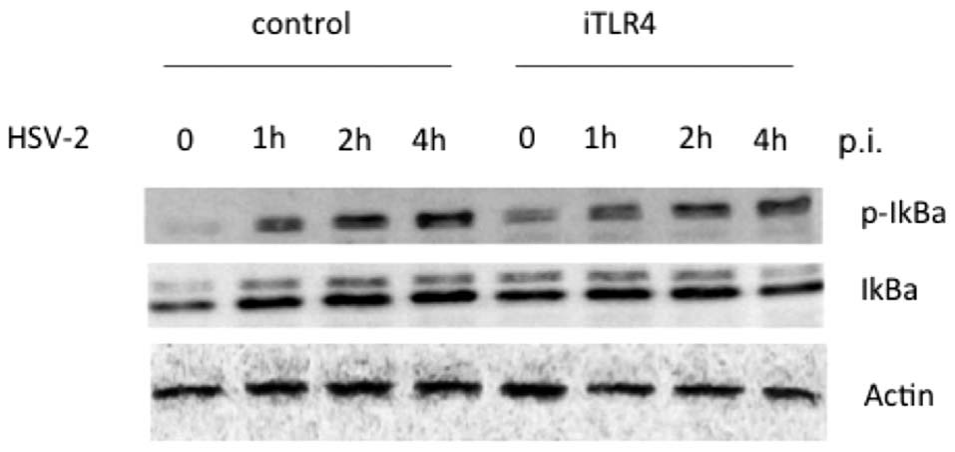
TLR4 mediates the activation of NF-kB signaling in response to HSV-2. HCE cells were transiently transfected with vector or iTLR4. Forty-eight hours after transfection, cells were infected with HSV-2 or mock-infected for 0 h, 1 h, 2 h and 4 h respectively. Cell lysates were analyzed by Western blotting with antibodies against p-IkB-α, total IkB-α and actin. Knockdown of TLR4 expression decreased phosphorylated IkB-α. The experiment was repeated three times, the representative images from one experiment are shown.

We have shown that HSV-2 infection activates NF-kB reporter activity in HCE cells. This activity requires cooperation of TLR4, and downstream adaptors as well as phosphorylated signaling components. We asked whether these activities correlate with the balance of inhibitor of NF-kB (IkB- α) and NF-kB. Western blot data in Figure 5 demonstrated the increased phosphorylated IkB- α after HSV-2 infection, while the total IkB- α remained relatively stable as actin (low panels). We also noted that the IkB- α was rapidly phosphorylated at 1 hrs upon HSV-2 infection and this inactive inhibitory form of NF-kB further increased at 2 and 4 hrs p.i.. This suggested that released inhibition of NF-kB (high levels of phosphorylated IkB- α) correlated the NF-kB activity. Most importantly, knockdown of TLR4 significantly impaired virus-induced phosphorylation of IkB- α at 1, 2, and 4 hrs after viral infection compared to 0 hr point (right part of the Figure 5). Thus, our data showed that HSV-2 induced activation of NF-kB signaling in human natural host HCE cells is TLR4 dependent.

### Adaptors, MyD88/Mal, are required for TLR4-dependent expression of cytokines induced by HSV-2

**Figure 6:**
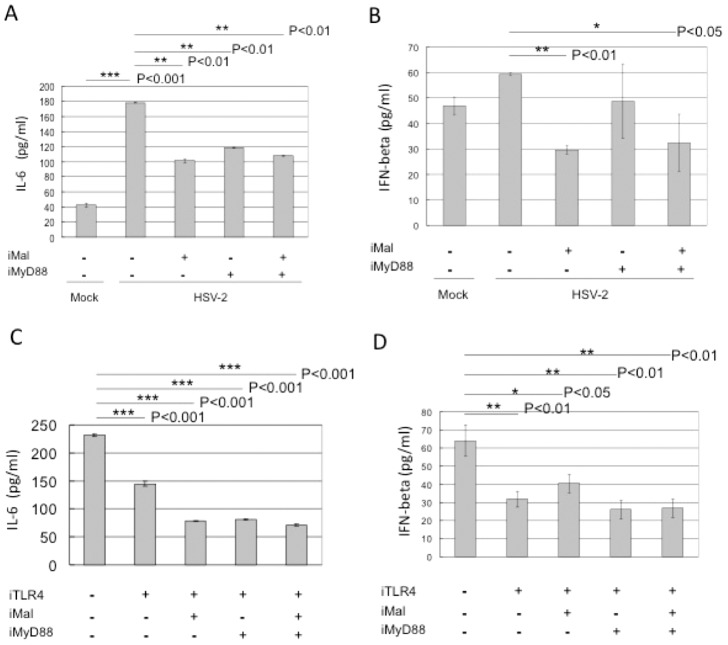
HSV-2 induces MyD88/Mal mediated cytokines in HCE cells. (A) and (C). MyD88/Mal-dependent IL-6 expression by HSV-2. HCE cells were transfected with negative control siRNA duplexes or iMal/iMyD88 (A) or together with iTLR4 (C). At 48 h after transfection, HCE cells were infected with HSV-2 at 3 MOI or mock-infected. Cell-free supernatants were collected at 4 h post-infection for measurement of IL-6 secretion by ELISA analysis. Each assay was performed in triplicate. Results are reported as means ± SD. ** indicates p<0.01, and *** indicates p<0.001. (B) and (D) MyD88/Mal-dependent IFN-β expression by HSV-2. Cell-free supernatants from above cell cultures were collected at 1 h post-infection for measurement of IFN-β secretion by ELISA analysis. Each assay was performed in triplicate. Results are reported as means ± SD. * indicates p<0.05, and ** indicates p<0.01.

Lastly, we wanted to assess the role of adaptor molecules (MyD88/Mal) in production of cytokines in HCE cells in response to HSV-2. HCE cells were transfected with siRNA duplexes of iMal, iMyD88 or iMal/iMyD88. After 48 h of transfection, cells were infected with HSV-2 at 3 MOI or mock-infected. The supernatants were collected at 1 h p.i. or 4 h p.i. and measured for the secretion of IL-6 and IFN- β by ELISA. In Figure 6A, 42.74 ± 2.45 pg/ml of IL-6 were detectable in HCE cells with mock infection and scramble siRNA transfection, HSV-2 infection resulted in a dramatic increase (178.2 ± 0.52 pg/ml) of IL-6 (p<0.001), while knockdown of either MyD88, or Mal alone or both significantly decreased expression of IL-6 at about 34–43% (p<0.01). In Figure 6C, we showed about 37–69% decrease in IL-6 expression with knockdown of TLR4 alone or together with either MyD88 or Mal or both (p<0.001). A similar pattern of the changes with expression of IFN- β was observed (p<0.05 or 0.01)(Figure 6B and 6D). These data indicated that adaptors, MyD88 and Mal, are required for TLR4-dependent secretion of inflammatory cytokines and IFN- β in response to HSV-2 in HCE cells.

## Discussion

**Figure 7:**
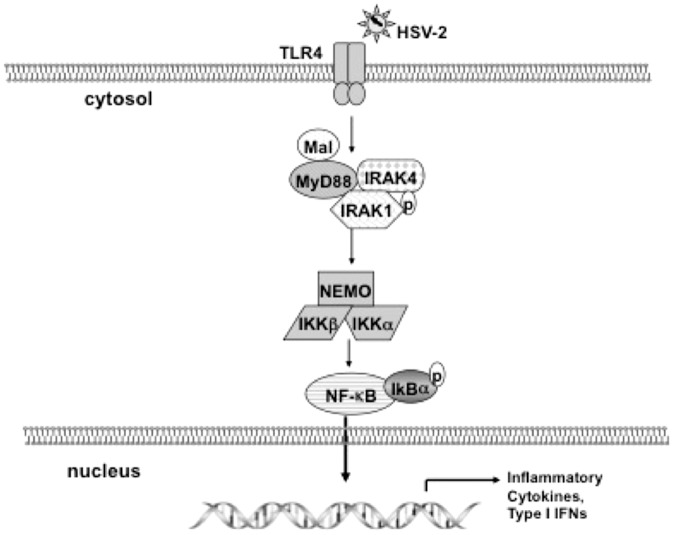
The proposed model that HSV-2 induces TLR4-MyD88/Mal-NF-kB signaling and innate immune response in HCE cells. HSV-2 infection induces TLR4 dependent signaling, adaptors molecules (MyD88/Mal) and phosphorylated IRAK1 are involved in the signal transduction to downstream NF-kB. The NF-kB signaling is activated by the phosphorylation and degradation of IkB-α and then active form of NF-kB is translocated into nucleus, thereby regulating the production of inflammatory cytokines and IFN-β.

In the current study, we demonstrated for the first time that TLR-4/MyD88/Mal/NF-kB signaling axis was involved in HSV-2 infection in its natural host cells (Figure 7). It has been shown that multiple TLRs are involved in recognition of different HSV strains and contribute to the immune response to HSV infection [12]–[16]. However, it turns out very different results from these studies since they used immuno-competent cells or mice models. The underlying questions that warrant our attentions are: (i) HSV does not directly infect dendritic cells (DCs) [9]; (ii) Different types of cells are likely to have different immune responses to the same virus; (iii) There exist inherent differences between rodent and human. Recently, a Canadian research group emphasized the importance of the human genital epithelium in the study of innate immune response to HSV-2 [7]. Although they investigated the susceptibility and antiviral activity of primary human female genital epithelium to HSV-2 by using TLR ligands[16], [22], the TLR-mediated innate signaling pathways in response to HSV-2 in the human genital epithelium remains unclear.

Previously, we have shown that HSV-2 activates NF-kB activation and TLRs potentially contributes to the inflammatory response in HCE cells [24]. In this study, we found that HSV-2 infection activates TLR4-dependent NF-kB reporter activity in HCE cells and knockdown TLR4 and TLR4 antagonist decreased NF-kB activity (Figure 1 and 2). To the best of our knowledge, this is the first report demonstrating the activation of TLR4-dependent signaling in response to HSV-2 infection. Most recently, Lagos et al. reported that infection of human lymphatic endothelial cells (LEC) with human Kaposi Sarcoma herpesvirus (KSHV) suppresses TLR4 expression and leads to immune escape [27]. Their work indicated for the first time the critical role of TLR4 in mediating innate response during primary infection and lytic replication of the latent DNA virus. TLR9 is thought to be the typically sensor of DNA viruses such as HSV. However, recent studies using natural DNA suggest that both CpG content and the level of methylation of the motif strongly affect the ability of DNA to activate TLR9 [28]. Although human platelets express TLR 1-9[29], TLR9 expression is mainly restricted to B cells and plasmacytoid DCs in humans[30]. Therefore, TLR9 is only important for the host immune response to pathogens that can reach the lymphoid organs or blood [20], [30]. TLR3 is also known to be activated by the replication intermediate double-stranded RNA (dsRNA) of HSV[20]. However, we could not detect TLR3 expression in human cervical epithelial cells (HCE) (data not shown). Other study reported that epithelial and dendritic cells use TLR3-independent pathways to prevent dissemination of HSV-1 [31]. It has been shown that TLR2 plays an important role in the inflammatory response to HSV-1 infection [14], [32], but not all strains of HSV-1 activate TLR2 [12]. It was proposed that HSV envelope protein can be recognized at the cell surface not only by TLR2 but TLR4 as well [33]. The innate immune response to HSV infection is complex since different virus strains have different cellular tropisms and the host immune response to viral infection is also cell-type specific.

Among all the members of TLRs family, TLR4 is unique because four adaptor molecules can be utilized to sort and determine the specific of its downstream signaling. TLR4 is capable to activate MyD88-dependent and TRIF-dependent signaling in macrophages to LPS stimulation [10], [34]–[36]. However, the exact TLR4-dependent signaling pathway triggered by Herpes virus is still unknown. Georgel et al. reported that Vesicular stomatitis virus (VSV) glycoprotein G activates TLR4-dependent pathway in mouse DCs [33]. They demonstrated that only adaptor TRAM (partial TRIF) is needed for TLR4 signaling which does not stimulate NF-kB activation. However, our previous study has shown that the activation of NF-kB is strictly required for the production of both IL-6 and IFN- β induced by HSV-2 in HCE cells [24]. Therefore, we wonder what components in HSV-2 induced TLR4-dependent signaling present in human cervical epithelial cells. Our results showed that adaptor molecules MyD88 and Mal are both required for TLR4-dependent signaling in response to HSV-2 (Figure 7). The key downstream protein of Mal/MyD88 is IRAK1, its phosphorylation activates TAK1 and IKK complex [10]. Knockdown of TLR4 clearly decreased the phosphorylation of IRAK1 (Figure 4). NF-kB is the central player in cellular immediate-early pathogen immune responses [37]. Silenced expression of TLR4 by RNA interference impaired the NF-kB signaling, this is due to decreased phosphorylation of IkB- α compared to the control (Figure 5). However, knockdown of TLR4 could not completely abolish the activation of IRAK1 and NF-kB in response to HSV-2 in HCE cells. One possibility is that other pattern-recognition receptors (PRRs), for example TLR2, are also activated by HSV-2. We have observed that expression of TLR2 is up-regulated in a later phase upon HSV-2 infection (data not shown). Probably they are triggered and function sequentially in HCE cells during HSV-2 infection course. Taken together, our results identified the TLR4-dependent Mal/MyD88-IRAK1-NF-kB axis in response to HSV-2 in its natural host cells.

Subsequently production of type I IFN and pro-inflammatory cytokines following TLR signaling activation will aid to abrogate further viral infection. Our data demonstrate that knockdown of TLR4 significantly decreased the secretion of IL-6 and IFN- β in response to HSV-2 infection (Figure 6C, 6D). Silenced expression of Mal/MyD88 also suppressed the production of IL-6 (Figure 6A), but showed partially reduced effect on the production of IFN- β (Figure 6B). Although we found the secretion of both IL-6 and IFN- β are dependent on NF-kB in HSV-2 infected HCE cells [24]. Different patterns of Type I IFN production can be induced through different PRRs signaling in response to viral infection [38]. Type I IFN is so critical for the antiviral activity of the epithelial cells that it is regulated by multiple factors. Besides of transcription factors IRF3 and ATF-2/c-Jun, IRF7 is also involved in regulating IFN-β expression during TRIF-signaling [39]. We could not rule out the possibility that adaptor molecules TRIF/TRAM are also involved in TLR4-signaling in response to HSV-2 in HCE cells. In this case, NF-kB is only part of regulators for IFN- β functioning in the branch pathway TLR4-Mal/MyD88-IRAK1. Moreover, type I IFN is thought to be induced only in the intracellular compartments [40], it is worthwhile to investigate the exact location and time point that adaptors pairs Mal/MyD88 and TRAM/TRIF switch to function in the further study.

In summary, our data demonstrate that HSV-2 infection activates TLR4-dependent innate immune response in human cervical epithelial cells. The two adaptor molecules Mal and MyD88 of TLRs signaling pathways are also required for the HSV-2 induced TLR4 signaling pathway. Our results reveal for the first time the pathway via TLR4-Mal/MyD88-IRAK1-NF-kB axis in response to HSV-2 infection in its primary infected natural host cells.

## Methods

### Cells and virus

Human cervical epithelial (HCE) cells immortalized by hTERT were the gift from Dr. X. Liu in Department of Pathology of Georgetown University as described in the early studies [41],[42], [24]. HCE cells were grown in keratinocyte serum-free medium (K-SFM) (Invitrogen) supplemented with the provided 50 µg/ml bovine pituitary extract, 0.1 ng/ml recombinant epidermal growth factor, and 1% penicillin/streptomycin (Invitrogen).Vero cells (ATCC) were maintained in complete Dulbecco's modified Eagle's medium (DMEM) supplemented with 10% fetal bovine serum (FBS) and 1% penicillin/streptomycin. Cells were incubated at 37°C in a humidified incubator containing 5% CO_2_. Herpes simplex virus 2 (HSV-2, G strain) was obtained from American Type Culture Collection (ATCC). All stocks of HSV-2 were propagated and tittered using a standard plaque assay in Vero cells. For virus infection, HCE cells were infected with HSV-2 at a multiplicity of infection (MOI) of 3. At the indicated time, cells were lysed for Western blot analysis or luciferase assay, and conditioned media were collected for cytokine measurements by ELISA.

### Antibodies, TLR4 antagonist and plasmids

Rabbit polyclonal antibodies against TLR4, IkB-α, IRAK1 and phospho-IRAK1 were obtained from Santa Cruz Biotechnology (Santa Cruz, CA). A rabbit polyclonal antibody against phospho-IkB-α was purchased from Cell Signaling Technology (Cambridge, MA). Mouse monoclonal antibodies against actin, HA, GFP were purchased from Santa Cruz Biotechnology (Santa Cruz, CA). A mouse monoclonal antibody against Flag was obtained from Sigma-Aldrich (St. Louis, Mo). Eritoran tetrasodium was obtained from Eisai Ins. The constructs pcDNA3-YFP-TLR4, pcDNA3.1-N1, pFlag-CMV-hMD2, pCMV-HA-MyD88 and pcDNA3-Mal-GFP were obtained from Addgene (Cambridge, MA). The NF-kB firefly luciferase reporter plasmid was obtained from Clontech. The shRNA Construct specific for TLR4 and shRNA pGFP-V-RS Vector were purchased from ORIGENE (Rockville, MD). The siRNA duplexes specific for human MyD88 and Mal/TIRAP were obtained from Dharmacon (Chicago, IL).

### Transfection

Cells were plated in a 6-well plate or 24-well plate one day before transfection and were grown to 50% confluence. Cells were transfected with Lipofectamine 2000 (Invitrogen) and harvested for total RNA or cell-lysate after 24 h or 48 h. The over-expression of genes was determined by Western blot analysis. The knock-down efficiency of genes was confirmed by Western blot analysis or regular RT-PCR.

### Reverse Transcriptase-PCR

Total RNA from 2 × 10^6^ HCE cells was extracted with RNApre pure cell RNA kit containing DNase I (TIANGEN Biotech, Beijing). Two microgram total RNA was reverse transcribed with random primer (Promega). Specificity of RT-PCR was controlled with no-template as well as no-reverse transcriptase samples. Results are normalized to the housekeeping gene GAPDH. The following primers were used: GAPDH forward: 5′-CTCAGACACCATGGGGAAGGTGA-3′, Reverse: 5′-ATGATCTTGAGGCTGTTGTCATA-3′; hMyD88 forward:5′-GGCTGCTCTCAACATGCGA-3′, reverse: 5′-TGTCCGCACGTTCAAGAACA-3′; hMal forward: 5′-CTCACAGGACAGCCCACTAC-3′, reverse: 5′-ACGAAAGCCACCATCAGG-3′.

### Western blot analysis

At indicated time points, HCE cells were harvested with modified radioimmuno-precipitation assay (RIPA) buffer (50 mM Tris-Cl, 150 mM NaCl, 2 mM EDTA, 1% Nonidet P-40, 0.5% Nadeoxycholate, and 0.1% SDS [pH 7.4]) with protease inhibitors (Sigma). The cell lysates were centrifuged at 15,000 rpm for 20 min at 4°C and the supernatants were collected and a BCA(Beyotime) protein assay was performed. Fifty of total extract was subjected to electrophoresis on 12% SDS-PAGE gel, and transferred onto polyvinylidene difluoride (PVDF) membranes (Millipore). Membranes were blocked in 5% milk-TBST buffer at 4°C. The membranes were probed with specific antibodies described in Antibodies and plasmids. Blots were developed using an enhanced chemiluminescence reagent (Beyotime).

### Luciferase reporter assays

HCE cells were seeded at a density of 1 × 10^4^ per 24-well plate and co-transfected with 500 ng of the NF-kB luciferase reporter plasmid and 2 ng of Renilla luciferase plasmid using Lipofectamine 2000 (Invitrogen) according to the manufacturer's instructions. For TLR4 and adaptor molecules augmentation assays, 40 nanograms of TLR4/hMD2/Mal/MyD88 or empty vector constructs were also co-transfected. The transient transfected cells were infected with HSV-2 at 3 MOI the following day and were harvested 4 hrs post-infection (p.i.). For TLR4 and adaptor molecules ablation assays, 40 nanograms of shRNA-TLR4 (refer to iTLR4) or a final concentration of 40 nM of siRNA duplexes specific for Mal or MyD88 (Dharmacon) were also co-transfected. The transient transfected cells were infected with HSV-2 at 3 MOI at 48 h after transfection and were harvested 4 hrs post-infection (p.i.). Luciferase activity was measured using a Dual-Glo luciferase reporter assay system (Promega) on a GLOMAX 20/20 luminometer (Promega). NF-kB firefly luciferase activity was normalized to Renilla luciferase activity. The results are shown as means ± SD of triplicate wells and expressed as fold induction. Each experiment was repeated three times.

### Enzyme-linked immunosorbent assay (ELISA)

HCE cells were cultured at 1 × 10^4^ cells/well in flat-bottom 24-well plates for 16–24 h, transient transfected with negative control siRNA duplexes or siRNA duplexes for iMal or iMyD88, with or without shRNA-TLR4. At 48 h after transfection, HCE cells were infected with HSV-2 at 3 MOI. Cell-free supernatants were collected at 1 hrs or 4 hrs p.i. for ELISA analysis. The secretion of IL-6 and IFN-β were measured according to the manufacturer's instructions (R&D Systems, Abingdon, UK). The optical density was measured using a Bio-Kinetics microplate reader (Bio-Tek Instruments). Each assay was performed in triplicate. Results are reported as means ± SD.

### Statistical analysis

Two-way ANOVA, followed by the Bonferroni posttest, was used for analysis of the data by software Prism 5.0 (GraphPad Software, La Jolla, CA). For each test, differences were considered significant at p<0.05, and data are shown as mean ± SD. All of the experiments were reproducible and carried out in duplicate. Each set of experiments was repeated at least three times.
